# PEMD方案治疗初治早期非上呼吸道消化道或晚期ENKTL患者的有效性及安全性分析

**DOI:** 10.3760/cma.j.cn121090-20241120-00463

**Published:** 2025-02

**Authors:** 心怡 张, 凯欣 杜, 浩睿 申, 佳竹 吴, 悦 李, 华 尹, 莉 王, 金花 梁, 建勇 李, 卫 徐

**Affiliations:** 南京医科大学第一附属医院（江苏省人民医院）血液科，南京 210029 The First Affiliated Hospital of Nanjing Medical University（Department of Hematology, Jiangsu Province Hospital）, Nanjing 210029, China

**Keywords:** 淋巴瘤，结外NK/T细胞, 培门冬酶, 生存分析, Lymphoma, extranodal NK/T cell, Pegaspargase, Survival analysis

## Abstract

**目的:**

探讨PEMD（培门冬酶+依托泊苷+甲氨蝶呤+地塞米松）方案治疗初治早期非上呼吸道消化道或晚期结外NK/T细胞淋巴瘤（ENKTL）患者的疗效及安全性。

**方法:**

回顾性分析纳入2016年1月至2022年12月于南京医科大学第一附属医院血液科诊断为初治早期非上呼吸道消化道或晚期ENKTL，且接受PEMD方案诱导化疗的38例患者。采用Kaplan-Meier法计算无进展生存（PFS）和总生存（OS）率，采用Log-rank检验比较组间生存差异，进行生存和预后因素分析。

**结果:**

男30例（78.9％），女8例（21.1％）；中位年龄48（26～72）岁。7例（18.4％）年龄>60岁，7例（18.4％）美国东部肿瘤协作组（ECOG）体能评分>1分，20例（52.6％）LDH水平升高，37例（97.4％）出现鼻部以外的结外组织受累，37例（97.4％）Ann Arbor分期为Ⅲ～Ⅳ期。患者中位治疗5（1～6）个周期，中位随访时间为60（24～101）个月。所有患者治疗结束时的总有效率为52.7％。2年和4年的PFS率分别为34.2％（95％ *CI* 22.0％～53.2％）和25.5％（95％ *CI* 14.7％～44.4％），2年及4年OS率分别为50.0％（95％ *CI* 36.4％～68.7％）及45.5％（95％ *CI* 31.4％～65.7％）。ECOG体能评分>1分［*HR*＝3.711（95％ *CI* 1.494～9.218），*P*＝0.005］、骨髓浸润［*HR*＝2.251（95％ *CI* 1.026～4.938，*P*＝0.043）］及PINK-E评分3～5分［*HR*＝2.350（95％ *CI* 1.009～5.476），*P*＝0.048］为PFS的危险因素，多因素分析ECOG体能评分>1分为PFS的独立危险因素［*HR*＝7.971（95％ *CI* 2.222～28.591），*P*＝0.001］。安全性评估显示，主要的不良反应为贫血（31例，81.6％）。

**结论:**

PEMD方案对治疗初治早期非上呼吸道消化道或晚期ENKTL患者是安全有效的。

## Abstract (en)

**Objective:**

To evaluate the efficacy and safety of the pegaspargase, etoposide, methotrexate, and dexamethasone（PEMD）regimen in patients with early-stage nonupper respiratory digestive tract or advanced extranodal natural killer/T-cell lymphoma（ENKTL）.

**Methods:**

This retrospective analysis included 38 patients with newly diagnosed early-stage non-upper respiratory digestive tract or advanced ENKTL who received PEMD regimen for induction chemotherapy at the First Affiliated Hospital of Nanjing Medical University from January 2016 to December 2022. Survival outcomes and prognostic factors were examined by Kaplan-Meier, and the Log-rank test was used to compare survival.

**Results:**

The study population had a median age of 48 years（range, 26–72 years）and included 30 males（78.9％）and 8 females（21.1％）. 7 patients' age >60 years（18.4％）. The Eastern Cooperative Oncology Group（ECOG）performance score was >1 in 7 patients（18.4％）; 20 patients（52.6％）had elevated lactate dehydrogenase levels; and 37 patients（97.4％）exhibited extranodal involvement. Using the Ann Arbor staging system, 37 patients（97.4％）were classified as stage Ⅲ–Ⅳ. The median number of treatment cycles was 5（1–6）, and the median follow-up duration was 60 months（24–101 months）. Interim efficacy assessment revealed an overall response rate of 52.7％. At 2 and 4 years, the progression-free survival（PFS）rates were 34.2％（95％ *CI* 22.0％–53.2％）and 25.5％（95％ *CI* 14.7％–44.4％）, respectively, and the overall survival rates were 50.0％（95％ *CI* 36.4％–68.7％）and 45.5％（95％ *CI* 31.4％–65.7％）, respectively. The risk factors for worse PFS were ECOG performance score >1［*HR*＝3.711（95％ *CI* 1.494–9.218）, *P*＝0.005］; bone marrow infiltration［*HR*＝2.251（95％ *CI* 1.026–4.938）, *P*＝0.043］; and Prognostic Index for Natural Killer/T-Cell Lymphoma score of 3–5［*HR*＝2.350（95％ *CI* 1.009–5.476）, *P*＝0.048］. Multivariate analysis identified ECOG performance score >1 as an independent risk factor for PFS［*HR*＝7.971（95％ *CI* 2.222–28.591）, *P*＝0.001］. The main adverse effect was anemia in 31 patients（81.6％）.

**Conclusion:**

The PEMD regimen was safe and effective for patients with newly diagnosed early-stage non-upper respiratory digestive tract or advanced ENKTL.

结外NK/T细胞淋巴瘤（ENKTL）是一种侵袭性非霍奇金淋巴瘤，在亚洲和拉丁美洲发病率较高，且70％以上的ENKTL患者为早期[1]。由于ENKTL表达高浓度的多药耐药P-糖蛋白，导致该疾病患者对蒽环类药物产生耐药。因此，ENKTL患者对以蒽环类药物为基础的方案，如CHOP（环磷酰胺+多柔比星+长春新碱+泼尼松）方案的反应较差[2]。目前，基于门冬酰胺酶的化疗方案被广泛应用于ENKTL患者的治疗，包括改良SMILE（地塞米松+甲氨蝶呤+异环磷酰胺+左旋门冬酰胺酶+依托泊苷）方案、P-GEMOX（培门冬酶+吉西他滨+奥沙利铂）方案和DDGP（地塞米松+顺铂+吉西他滨+培门冬酶）方案，这些方案在临床上显示出了一定的疗效，特别是在早期ENKTL患者中可获得长期生存，但早期非上呼吸道消化道或晚期患者预后仍然较差[3]–[4]。

本中心既往前瞻性探索了PEMD（培门冬酶+依托泊苷+甲氨蝶呤+地塞米松）方案在35例晚期ENKTL患者中的疗效和安全性[5]，总有效率（ORR）为75％（32/35）、完全缓解（CR）率为47％（15/35），4年无进展生存（PFS）率和总生存（OS）率为44％（95％ *CI* 25％～63％）和51％（95％ *CI* 32％～70％）。本研究中，我们回顾性分析PEMD方案在本中心38例初治早期非上呼吸道消化道或晚期ENKTL患者中的有效性及安全性。

## 病例与方法

1. 病例：研究纳入2016年1月至2022年12月于南京医科大学第一附属医院血液科新诊断为ENKTL的38例患者。纳入标准如下：①根据2016年世界卫生组织造血和淋巴组织肿瘤病理学分类诊断为ENKTL[6]；②Ann Arbor分期为早期非上呼吸道消化道或晚期；③接受PEMD方案进行诱导化疗，并进行有效评估；④人口统计学数据、基线临床特征和实验室检验资料完整。本研究经南京医科大学第一附属医院伦理委员会批准（伦理号：2022-SR-761）。

2. 资料收集：从医院电子病历系统收集了基线人口统计学数据和临床特征，包括性别、年龄、美国东部肿瘤协作组（ECOG）体能评分、Ann Arbor分期、累及部位、PINK和PINK-E评分、实验室检查结果、影像学资料（治疗过程中所有^18^F-氟代脱氧葡萄糖PET-CT显像资料）。

3. 治疗方案：PEMD方案具体用药如下：甲氨蝶呤以3.0 g/m²的剂量在第1天静脉滴注，持续6 h；依托泊苷以100 mg/m²的剂量在第2至4天静脉滴注；地塞米松在第1至4天以40 mg的剂量静脉滴注；培门冬酶在第2天以2 500 U/m²的剂量肌肉注射。每3周为1个周期，拟进行4～6个周期的PEMD方案治疗。

4. 疗效评估：于以下时间点进行疗效评估：3个周期治疗结束后、末次治疗（4～6个周期）结束后、治疗结束后的2年内每3个月1次。疗效分为CR、部分缓解（PR）、疾病稳定（SD）和疾病进展（PD）。ORR为CR率和PR率之和。

5. 安全性评价：根据《常见不良反应事件评价标准（CTCAE）》5.0版，对患者每个周期用药后发生的不良事件（AE）进行分级。

6. 随访：通过住院病历、门诊就诊记录和电话对所有患者进行随访。随访截止日期为2024年11月8日。随访结局事件包括PFS和OS。OS期定义为从初始治疗至任何原因导致死亡或随访终点的时间。PFS期定义为从初始治疗至出现复发或进展的时间。

7. 统计学处理：患者的临床特征、疗效和AE等资料采用描述性统计分析，计数资料以例数（百分比）表示，计量资料以*M*（范围）表示。采用SPSS 26.0和GraphPad Prism 9软件进行统计学分析。采用Kaplan-Meier法计算PFS和OS，组间比较采用Log-rank检验，单因素与多因素分析采用Cox等比例风险回归模型。*P*<0.05为差异具有统计学意义。

## 结果

1. 临床特征：38例患者的基线临床特征如表1所示，中位年龄48（26～72）岁。其中有2例患者因缺失基线EBV-DNA数据，无法进行基线EBV-DNA定性分析及PINK-E分层。在37例（97.4％）出现鼻部以外的结外受累患者中，胃肠道和骨是最常受累的部位，均为11例（30.6％）；骨髓、肾上腺和皮下组织则各有10例（27.8％）患者受累；其次依次为肺（5例，15.5％）、女性生殖系统（5例，15.5％）、肾（4例，10.8％）、胰腺（4例，10.8％）、肌肉（4例，10.8％）、胸膜（3例，8.1％）及心脏（2例，5.4％），另有5例（15.5％）其他部位受累。

**表1:** 38例结外NK/T细胞淋巴瘤患者的基线临床特征

临床特征	例（％）
年龄>60岁	7（18.4）
男性	30（78.9）
ECOG体能评分>1分	7（18.4）
LDH>正常值	20（52.6）
鼻部以外结外组织受累	37（97.4）
骨髓浸润	11（28.9）
鼻型	21（55.3）
有远处淋巴结转移	24（63.2）
Ann Arbor分期为Ⅲ～Ⅳ期	37（97.4）
PINK评分	
0～1分	9（23.7）
2～4分	29（76.3）
PINK-E评分^a^	
0～1分	3（8.3）
2分	11（30.6）
3～5分	22（61.1）
EBV-DNA阳性^a^	28（77.8）

**注** ECOG：美国东部肿瘤协作组；PINK：NK/T细胞淋巴瘤预后指数（年龄、Ann Arbor分期、原发部位、是否远处淋巴结受累）；PINK-E：PINK+循环EBV-DNA是否阳性；EBV：EB病毒；^a^2例患者缺少EBV-DNA数据，拥有PINK-E评分及EBV-DNA数据的患者总数为36例

2. 疗效评价：患者接受PEMD方案中位治疗5（1～6）个周期。中位随访时间为60（24～101）个月。治疗结束后评估：ORR为52.7％，其中17例患者获得CR（44.7％），3例患者获得PR（7.9％）。在治疗结束时获得CR的17例患者中，仅3例患者接受了自体造血干细胞移植，4例患者治疗结束后接受放疗、1例患者治疗结束后口服西达本胺维持治疗、1例患者治疗结束后使用PD-1单抗维持治疗，其余患者因自身原因在治疗结束后未进行后续治疗（其中有4例患者在随访过程中出现PD后接受了二线方案治疗）。

3. 预后分析：38例患者的2年及4年PFS率分别为34.2％（95％ *CI* 22.0％～53.2％）和25.5％（95％ *CI* 14.7％～44.4％），2年及4年OS率分别为50.0％（95％ *CI* 36.4％～68.7％）和45.5％（95％ *CI* 31.4％～65.7％）。单因素分析，ECOG体能评分>1分［*HR*＝3.711（95％ *CI* 1.494～9.218），*P*＝0.005］、骨髓浸润［*HR*＝2.251（95％ *CI* 1.026～4.938），*P*＝0.043］及PINK-E评分3～5分［*HR*＝2.350（95％ *CI* 1.009～5.476），*P*＝0.048］为PFS的危险因素（表2）。将单因素分析中与PFS显著相关的因素纳入多因素分析，结果显示ECOG体能评分>1分为PFS的独立危险因素［*HR*＝7.971（95％ *CI* 2.222～28.591），*P*＝0.001］。

**表2:** 关于接受PEMD方案治疗的38例早期非上呼吸道消化道及晚期结外NK/T细胞淋巴瘤患者生存的单因素分析

临床特征	无进展生存		总生存	
	*HR*（95％ *CI*）	*P*值	*HR*（95％ *CI*）	*P*值
年龄>60岁	1.213（0.489～3.006）	0.677	1.224（0.358～4.180）	0.747
男性	0.459（0.190～1.093）	0.078	0.527（0.201～1.383）	0.193
ECOG体能评分>1分	3.711（1.494～9.218）	0.005	1.328（0.440～4.003）	0.615
LDH>正常值	1.309（0.622～2.758）	0.478	1.081（0.449～2.604）	0.862
鼻部以外结外受累	–	0.439	–	0.368
骨髓浸润	2.251（1.026～4.938）	0.043	2.274（0.923～5.607）	0.074
鼻型	0.986（0.466～2.085）	0.970	0.681（0.283～1.639）	0.392
有远处转移	2.249（0.925～5.465）	0.074	2.485（0.898～6.877）	0.080
Ⅲ～Ⅳ期	–	0.439	–	0.555
PINK评分2～4分	2.186（0.826～5.788）	0.115	2.443（0.712～8.378）	0.156
PINK-E评分3～5分^a^	2.350（1.009～5.476）	0.048	2.224（0.857～5.876）	0.100
EBV-DNA阳性^a^	2.020（0.758～5.385）	0.160	1.749（0.582～5.250）	0.319

**注** PEMD：培门冬酶+依托泊苷+甲氨蝶呤+地塞米松；ECOG：美国东部肿瘤协作组；PINK：NK/T细胞淋巴瘤预后指数（年龄、Ann Arbor分期、原发部位、是否远处淋巴结受累）；PINK-E：PINK+循环EBV-DNA是否阳性；EBV：EB病毒；^a^2例患者缺少EBV-DNA数据，拥有PINK-E评分及EBV-DNA数据的患者总数为36例；–：例数较少无法计算

4. PEMD方案治疗ENKTL的安全性评估：如表3所示，PEMD方案最常见的血液学不良反应是贫血（31例，81.6％），最常见的非血液学不良反应为低白蛋白（21例，60.5％）。此外，最常见的3级以上不良反应为血小板减少（11例，28.9％）。

**表3:** 接受PEMD方案治疗的38例早期非上呼吸道消化道及晚期结外NK/T细胞淋巴瘤患者的不良反应［例（％）］

不良反应类型	不良反应	3级以上不良反应
血液学不良反应		
贫血	31（81.6）	2（5.2）
白细胞减少	19（50.0）	6（15.8）
血小板减少	24（63.2）	11（28.9）
非血液学不良反应		
感染	11（28.9）	3（7.9）
低白蛋白	23（60.5）	4（10.5）
低纤维蛋白原	21（55.3）	3（7.9）
肝功能损害	7（18.4）	2（5.2）
肾功能损害	2（5.2）	1（2.6）
腹泻	1（2.6）	1（2.6）

**注** PEMD：培门冬酶+依托泊苷+甲氨蝶呤+地塞米松

## 讨论

在既往报道过的病例中，非上呼吸道消化道的ENKTL患者预后明显较差[3]–[4]，此类患者除在PET-CT上明确显示仅有局限侵犯外，均需要接受与晚期ENKTL患者相同的治疗[7]。ENKTL具有侵袭性强、易耐药、预后差等特点，近年来针对其治疗方案有诸多临床研究。目前，含门冬酰胺酶的化疗方案是ENKTL最有效的全身化疗方案[8]。其中，SMILE方案是当前研究较多的晚期ENKTL治疗方案[8]–[10]。在接受SMILE治疗的初治晚期及复发/难治ENKTL患者中，2个周期化疗后的ORR为79％、CR率为45％；但该方案的骨髓抑制明显，有92％的患者出现了4级中性粒细胞减少症[10]。其他含门冬酰胺酶的方案，如AspaMetDex（左旋门冬酰胺酶+甲氨蝶呤+地塞米松）、MEDA（甲氨蝶呤+依托泊苷+地塞米松+门冬酰胺酶）以及P-GEMOX方案也已用于初治晚期ENKTL患者[11]–[13]，DDGP、COPE-L（环磷酰胺+依托泊苷+长春新碱+泼尼松+门冬酰胺酶）、PD-1单抗联合培门冬酶、PD-1单抗联合P-GEMOX方案及PD-1单抗联合放疗也取得不错疗效[14]–[19]，其中信迪利单抗联合P-GEMOX方案的ORR为100％，CR率为85％，2年PFS率和3年OS率分别为64％和76％[18]。

本中心既往前瞻性探索了PEMD方案在35例晚期ENKTL患者中的疗效和安全性，结果提示PEMD安全且有效[4]。因此，我们进一步探索PEMD方案在初治早期非上呼吸道消化道或晚期ENKTL患者的有效性及安全性。在本研究中，38例患者接受1～6个周期的PEMD方案治疗后，ORR为52.7％，其2年及4年PFS率分别为34.2％（95％ *CI* 22.0％～53.2％）及25.5％（95％ *CI* 14.7％～44.4％），2年及4年OS率分别为50.0％（95％ *CI* 36.4％～68.7％）及45.5％（95％ *CI* 31.4％～65.7％）。血液学不良反应是最常见的不良反应，非血液学不良反应主要表现为低白蛋白和低纤维蛋白原。以上提示PEMD方案对于治疗初治早期非上呼吸道消化道或晚期ENKTL患者具有一定的有效性，但整体的ORR和4年的PFS率和OS率低于前瞻性研究，主要可能与入组患者的临床特征更加高危有关。

近年来，研究者们还针对ENKTL的分子机制进行广泛研究，以求寻找更有效的治疗方案[20]。基于最新基因组测序（WGS/WES）、拷贝数变异（CNV）和转录组测序的联合研究显示，ENKTL可以分为3种不同分子亚型，包括TSIM、MB和HEA亚型；临床前研究发现，TSIM亚型患者对免疫抑制剂抗PD1抗体敏感、MB亚型患者对高三尖杉酯碱敏感、HEA亚型患者对组蛋白去乙酰化酶抑制剂敏感，此研究为上述靶向药物应用于ENKTL临床诊疗提供重要理论依据[21]。

由于本研究是回顾性分析，且为单中心研究，样本量较小，需前瞻性多中心研究且扩大样本量，以验证PEMD方案的有效性和安全性。此外，仍需要随机对照研究（以门冬酰胺酶为基础的化疗方案）来为本研究提供更强有力的循证医学证据。
